# Food Intolerances

**DOI:** 10.3390/nu11071684

**Published:** 2019-07-22

**Authors:** Caroline J Tuck, Jessica R Biesiekierski, Peter Schmid-Grendelmeier, Daniel Pohl

**Affiliations:** 1 Gastrointestinal Diseases Research Unit, Queen’s University, Kingston, ON K7L 2V7, Canada; 2 Department of Dietetics, Nutrition and Sport, La Trobe University, Melbourne 3086, Australia; 3 Allergy Unit, Department of Dermatology, University Hospital Zurich, 8091 Zurich Switzerland and Christine-Kühne Center for Allergy Research and Education CK-CARE, 7265 Davos, Switzerland; 4 Department of Gastroenterology, University Hospital Zurich, 8091 Zurich, Switzerland

**Keywords:** food intolerance, dietary therapy, food hypersensitivity, functional gastrointestinal disorders

## Abstract

Food intolerances are estimated to affect up to 20% of the population but complete understanding of diagnosis and management is complicated, given presentation and non-immunological mechanisms associated vary greatly. This review aims to provide a scientific update on common food intolerances resulting in gastrointestinal and/or extra-intestinal symptoms. FODMAP sensitivity has strong evidence supporting its mechanisms of increased osmotic activity and fermentation with the resulting distention leading to symptoms in those with visceral hypersensitivity. For many of the other food intolerances reviewed including non-coeliac gluten/wheat sensitivity, food additives and bioactive food chemicals, the findings show that there is a shortage of reproducible well-designed double-blind, placebo-controlled studies, making understanding of the mechanisms, diagnosis and management difficult. Enzyme deficiencies have been proposed to result in other food sensitivities including low amine oxidase activity resulting in histamine intolerance and sucrase-isomaltase deficiency resulting in reduced tolerance to sugars and starch. Lack of reliable diagnostic biomarkers for all food intolerances result in an inability to target specific foods in the individual. As such, a trial-and-error approach is used, whereby suspected food constituents are reduced for a short-period and then re-challenged to assess response. Future studies should aim to identify biomarkers to predict response to dietary therapies.

## 1. Introduction

Adverse food reactions are defined as any abnormal reaction following the ingestion of food. The different adverse reactions are described as food hypersensitivity, including food intolerance and food allergy, or food aversion, which is a psychological avoidance by Pavlovian conditioning of adverse reactions [1]. There are key pathophysiological differences between food allergy and food intolerance, resulting in different diagnostic strategies and therapeutic options and are divided according to having an immune basis or not. The definition of food intolerance is a non-immunological response initiated by a food or food component at a dose normally tolerated and account for most adverse food responses. Food allergy is an abnormal immune response to a food protein mediated by immunoglobulin E (IgE), non-IgE or mixed IgE/non-IgE immunological mechanisms. The prevalence of food allergy varies affecting 1–2% of adults and less than 10% of children [2]. In contrast, food intolerance is estimated to affect up to 20% of the population [3]. Despite food intolerance being so common worldwide, the diagnosis is often not straightforward and requires an understanding of the varied clinical presentation including severity and timing of symptom onset. This is further complicated by the various mechanisms of food intolerance that can exist, ranging from pharmacological (e.g. caffeine), to enzyme deficiencies (e.g. lactose malabsorption), to non-specific gastrointestinal (GI) functioning [2]. 

The aim of this narrative review was to critically evaluate and synthesize the key evidence and scientific understanding for the most common food intolerances. Each type of food intolerance assessed presents an overview of the food component and where it is found, a description of the induced response including any prevalence data, an overview of the mechanisms of how the food may induce its response and suggested diagnosis and management. Recent literature was analyzed via search databases including Medline, PubMed and Scopus to conduct this narrative review.

## 2. Sensitivities (Non-Allergic) 

### 2.1. FODMAPs 

Naturally occurring and present in a wide variety of foods are fermentable carbohydrates termed ‘FODMAPs’ (denoting fermentable oligo- di- mono-saccharides and polyols). This group of carbohydrates has been identified to trigger symptoms in patients with functional GI symptoms such as in irritable bowel syndrome (IBS) [4]. The low FODMAP diet has been extensively studied worldwide and shown good evidence for efficacy, is supported by meta-analyses [5,6] and has been incorporated into clinical guidelines [7]. An important factor that has allowed for reproducible results has been the establishment of food analysis techniques and subsequent detailed food composition data available [8,9,10,11,12,13]. The low FODMAP diet reduces intake of carbohydrate subgroups including (i) excess fructose present in foods such as honey, apples and mangoes, (ii) lactose present in milk and yoghurt (in the presence of lactase deficiency), (iii) polyols (largely comprised of sorbitol and mannitol) present in avocado and pears, iv) fructans present in wheat, onion and garlic, and (v) galacto-oligosaccharides present in legumes and nuts. Detailed and regularly updated food lists are available elsewhere [14]. While a wide range of foods are classified as high in FODMAP content, no one food group is excluded from the diet. Therefore, when the diet is properly implemented with suitable alternatives included, nutritional adequacy is largely maintained [15]. 

#### 2.1.1. FODMAP Induced Response

Consumption of a high FODMAP diet in sensitive individuals is thought to be associated with lower GI symptoms of abdominal pain, bloating, flatulence, and altered bowel habits. Randomized controlled trials using a low FODMAP diet have noted improvement in overall abdominal symptoms, pain, bloating, and bowel habit [4,16,17,18]. However, one study found significance in reducing abdominal pain, but not overall symptom control, although this may be an artifact of the study design [19]. Studies have noted that a low FODMAP diet can improve both diarrhea and constipation [4], although less data is available for the constipation predominant subtype. More recently, studies have suggested that FODMAPs may alter upper GI motility, affecting intra-gastric pressure [20] and may be useful in the management of functional dyspepsia [21]. Extra-intestinal symptoms have not been well studied with the low FODMAP diet, although fatigue has also been noted to improve [22]. Improvement in symptoms with the use of the low FODMAP diet in cohorts of IBS patients have been shown to vary between 50–80% [23]. This range is likely to be influenced by several factors including study design, patient selection and FODMAP intakes within habitual diets. 

#### 2.1.2. Proposed Mechanisms of FODMAP Induced Symptoms

Two main mechanisms of action were originally proposed for the effect of FODMAPs on symptom induction. Firstly, short-chain poorly absorbed carbohydrates present in the small intestinal lumen have an osmotic effect, increasing water delivery to the lumen. This mechanism has been confirmed whereby increased effluent output was shown in ileostomates given a high FODMAP diet [24], as well as higher osmotic volume noted in MRI studies [25,26]. Secondly, the delivery of rapidly fermentable carbohydrates to the colon leads to fermentation by colonic bacteria resulting in increased gas production. This mechanism has been confirmed via studies using MRI [25,26] and breath testing [22]. The combined effects of increased water delivery and gas in the lumen cause distention leading to pain and discomfort in patients with visceral hypersensitivity. These increased symptoms in susceptible individuals have been shown in carefully designed studies with adequate subject blinding [4,16]. It has also been shown that this effect is specific to those with visceral hypersensitivity, with minimal symptoms noted in healthy controls [4,26]. More recently, other mechanisms of action have been proposed such as potential of immune activation from high FODMAP foods due to significant reductions in urinary histamine [27] and pro-inflammatory cytokines [28] following a low FODMAP diet. However, further data is required to corroborate these findings and confirm their influence on symptomatic response.

#### 2.1.3. Diagnosis and Management of FODMAP Related Intolerances

The low FODMAP diet may be implemented following a diagnosis of IBS based on symptom criteria (Rome IV criteria), with adequate exclusion of organic disease [29]. Prior to commencement, a lactose breath test may be used to assess the need for lactose restriction. However, no other biomarkers or testing can provide reliable information regarding food tolerance to the various FODMAP subgroups. The low FODMAP diet should be implemented under guidance of a dietitian and is designed to be a three-phase diet. This includes a short-term (2–8 week) reduction in FODMAP intake [30], followed by strategic re-challenge to assess tolerance [31], and finally long-term maintenance where foods are only avoided as identified during re-challenge to maintain symptom control [32]. The United Kingdom National Institute for Health and Care Excellence (NICE) guidelines for adults with IBS suggest that the low FODMAP diet can be used as a second-line therapy following general lifestyle and dietary advice such as consumption of regular meals and avoidance of suspected trigger foods [33]. Certainly, the dietary advice should be tailored to the individual’s habitual diet and how their culture and cuisine may affect their FODMAP intake, highlighting the individual role of the dietitian in the implementation process.

As with any restrictive diet, the potential consequences of the dietary alterations during a low FODMAP diet should be considered. Firstly, high FODMAP foods are generally good sources of prebiotics, especially those containing oligosaccharides, and hence reduced intake may alter the microbiota profile. Short-term studies have suggested a low FODMAP diet may result in reductions in overall bacterial abundance, bifidobacteria and *Faecalibacterium prausnitzii* [34,35], although the long-term consequences on the microbiota following re-challenge is not known. Secondly, alterations in nutritional adequacy has been raised as a concern for patients utilizing the diet, with some studies showing reductions in calcium [34] and fibre [36] intakes during the restrictive phase of the low FODMAP diet and one study from the UK suggesting that those on the diet were less likely to meet the national dietary guidelines [37]. As with alterations to the microbiota, little is known of the long-term consequences of the diet on nutritional adequacy. One study with 6 month follow up showed that fibre intakes returned to pre-intervention levels following re-challenge [17], suggesting that the long-term consequences are likely to be minimal so long as re-challenge is undertaken. In addition to the need for long-term data, future studies should assess the ability of patients to be compliant with the low FODMAP diet. It is thought that dietitian guidance is key to the success of the diet [38], but further data is needed to understand dietary compliance, nutritional adequacy and effects on the microbiota profile both in the short- and long-term depending on mode of education (i.e., dietitian taught, physician taught or self-implemented). 

### 2.2. Wheat

The two parts of wheat relevant for discussion for food intolerance are the protein and carbohydrate fractions. Gluten is the main storage protein of wheat grains and is a complex mixture of hundreds of related but distinct proteins, mainly gliadin and glutenin. Similar storage proteins exist in rye, barley and oats, and are collectively referred to as “gluten”. Gluten contributes to the dough quality and is predominantly found in sources such as pasta, cake, pastries and biscuits, but can also be used as a binding and extending agent in processed foods. Other low molecular weight proteins found in wheat and related cereals are called α-amylase/trypsin inhibitors (ATIs), however these contribute less than 4% of the total protein content [39]. Another part of the protein component is wheat germ lectin agglutinin known for their binding action to expressed sugars [40]. The carbohydrate component (primarily fructan) of the wheat structure is discussed in the FODMAP section of this review. 

#### 2.2.1. Wheat Induced Response 

Outside of the auto-immune condition of coeliac disease and wheat allergy (both of which will not be discussed in this review), gluten is associated with GI symptoms including abdominal pain, bloating and bowel habit abnormalities, so-called ‘non-coeliac gluten/wheat sensitivity’ (NCG/WS). In addition to the GI response, there are various systemic manifestations reported to be associated with wheat intake, including disorders of the neuropsychiatric area such as “foggy mind”, headache, fatigue and dermatological and musculoskeletal symptoms (i.e., leg or arm numbness) [41,42]. Despite frequent self-diagnosis of NCG/WS, reproducibility of these symptoms is limited following a process of double-blind, placebo-controlled (DBPC) challenges [36,43], for example only 5% and 14% of two cohorts of self-reported NCG/WS patients showed symptomatic response to gluten [36,44]. Other DBPC trials using gluten powered challenges in IBS patients show conflicting results, showing symptom induction with gluten, however caution is warranted when drawing comparisons given study design differences (e.g., non-crossover) [45].

#### 2.2.2. Proposed Mechanisms of Wheat Related Intolerances

Due to the lack of reproducibility in clinical response, the mechanisms by which gluten causes symptoms within the food intolerance setting have been difficult to conclusively understand. Mechanistic studies have hinted at various roles for gluten’s action including, but not limited to, increased lipopolysaccharide-binding protein [46], increased eosinophils [47], both innate [48] and adaptive [49] immune activation, increased intestinal permeability and gut microbiota changes [50]. The evidence for mechanisms of ATIs remains in its infancy. ATIs activate innate immune cells via stimulation of toll-like receptor 4, which then induce the release of pro-inflammatory cytokines and chemokines [51]. Some in vitro and in vivo work has suggested that these ATIs may increase the gluten-specific T-cell response in coeliac disease [51]. It has been suggested that this inflammatory response may exacerbate inflammation at extra intestinal sites [52]. Wheat lectin agglutinin has shown in vitro that it can increase intestinal permeability by its epithelial damaging and immune effects [53]. The heterogeneity of results combined with consistent evidence indicative of the overlap between IBS and gluten-related disorders and high placebo and nocebo responses [36], means elucidating the underlying mechanisms of true wheat or gluten intolerance requires much more data.

#### 2.2.3. Diagnosis and Management of Wheat Related Intolerances 

Given there are no validated diagnostic biomarkers, the definition according to the scientific literature for NCG/WS is where subjects are responsive to a gluten-free diet and react to re-challenge with gluten compared to placebo [41]. This DBPC gluten re-challenge method is fundamentally complex and time-consuming to undertake, however critical to identifying and further examining the NCG/WS population. Care should be taken to ensure coeliac disease is definitively ruled out, especially where latent or subclinical coeliac markers of anti-tissue transglutaminase exist or in subjects with the coeliac associated genotype of HLA-DQ2/8. Clinical management, particularly for GI symptomatic improvement is likely to come from implementing the low FODMAP diet [36]. Furthermore, because of the coexistence of fructans and gluten in wheat, identifying the causative factor(s) is a crucial step in confirming which of the gluten-free or low FODMAP diets to instruct. The first randomised placebo-controlled trial to demonstrate that patients with NCGS appear to be intolerant to fructans not gluten [54] strengthens the argument for FODMAP (especially fructan) restriction as a first step. More attention to extra-intestinal sensations and wellbeing, including anxiety and depression, might inform why patients continue to follow a GFD and report to feel better.

### 2.3. Histamine 

Histamine is a biogenic amine that is present in the body but also in many foods. It is found in significant amounts in fermented and similar biogenic amines and can also be found in canned food, ready meals, semi-finished products or products that have been stored for a long time. The more perishable the food and the higher the protein content, the more important it is to consider food preparation and storage [55]. Table 1 outlines food groups containing histamine that have been reported to cause discomfort. 

#### 2.3.1. Histamine Induced Response 

Histamine intolerance can lead to unspecific GI symptoms and extra-intestinal symptoms, which occur mainly during and immediately following meals. Symptoms associated with histamine can occur in isolation or in combination (Table 2) [57,58].

#### 2.3.2. Proposed Mechanisms of Histamine Intolerance

Histamine intolerance results from an imbalance of accumulated or ingested histamine and the reduced ability to degrade histamine [59]. In healthy individuals, amine oxidases can quickly detoxify histamine ingested with food, while people with low amine oxidase activity run the risk of histamine toxicity. Diamine oxidase (DAO) is the main enzyme for the metabolism of ingested histamine. A second enzyme involved in the breakdown of histamine is histamine-N-methyltransferase (HNMT), a cytosolic protein that can only convert histamine in the intracellular space of cells. A disturbed or slowed histamine degradation due to reduced DAO activity and the resulting histamine excess may cause the symptoms mentioned above, particularly in cases of high intakes of dietary histamine. Genetic variants may also play a role [60]. 

Histamine occurs in different concentrations in animal and plant foods but is also produced and denatured by the body itself, for example in the intestinal microbiome. Histamine produced in the body seems to have less effect on histamine intolerance, except in situations such as mastocytosis [61] with an increased number of mast cells or the so-called mast cell activation syndrome (MCAS), in which spontaneous mast cell release due to still partially unknown factors leads to spontaneous histamine release in the body [62]. 

#### 2.3.3. Diagnosis and Management of Histamine Intolerance

Histamine intolerance remains an exclusion diagnosis, as there are no clear diagnostic criteria nor biomarkers for histamine intolerance. The diagnosis of histamine intolerance is made by a combination of criteria (Table 2) [58].

Other food intolerances, GI disease, IgE-mediated food allergies and underlying mastocytosis should be excluded before a diagnosis can be made. If histamine intolerance is still suspected, avoidance of histamine-rich foods for 4 to 6 weeks is undertaken. The measurement of DAO or histamine levels in the blood has been scientifically inconclusive and is not recommended for routine diagnosis or only after well-defined dietary regimens. A recent study has shown increased DAO after histamine restriction, reflecting dietary compliance [63]. Some genetic tests involved in the two main histamine degradation pathways, including DAO and HNMT are available on the market; however, evidence-based studies on their relevance in histamine intolerance are still lacking. On the other hand, the measurement of tryptase in serum can be helpful in obtaining evidence of the underlying mastocytosis. If the diagnosis is still unclear after all these steps, a DBPC oral histamine provocation may be the last evidential indication of diagnosis. Such an oral challenge requires a timely specific protocol with supplementary dietary and psychosomatic support.

**Figure 1:**
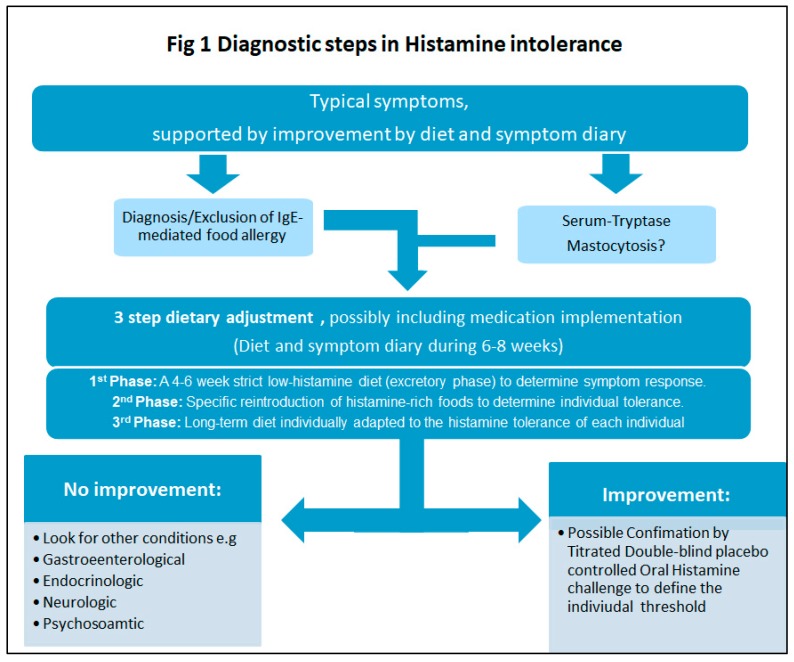
Diagnostic steps in Histamine intolerance. Modified by Reese et al. (2017) [58].

A low histamine diet is the first priority in management, which includes a three-stage diet change as shown in Figure 1. As a supporting measure, the enzyme DAO can be taken orally approximately one hour before histamine-rich food is consumed. In some cases, regular use of H1-blockers (antihistamines) may also help relieve symptoms. Long-term restriction should be avoided and should only be followed in close consultation with the doctor and/or nutritionist/dietitian. If no symptom improvement is observed, all foods should be gradually reintroduced.

### 2.4. Food Additives and Bioactive Food Chemicals

Thousands of different food additives are used throughout the food industry for various functions, especially to preserve food and improve taste or appearance. They are generally synthetic and natural substances that cannot be consumed alone as food themselves. Food additives are classified based on their function and property including preservatives, flavors, emulsifiers, thickeners, humectants, firming agents and flavor enhancers. A small number of additives have been implicated in IgE-mediated or other immunological or non-immunological adverse reactions.

A low food chemical diet encompasses restriction of a wide range of bioactive food additives and preservatives that occur naturally or are artificially added to foods (Table 3). The low food chemical diet, also referred to as “The Elimination Diet“ was originally conceptualized by the Royal Prince Alfred Hospital Allergy Unit in Sydney, Australia [64] and is thought to reduce GI symptoms, although data from controlled trials is lacking. While some food composition data is available, the food chemical content of foods is likely to be influenced by growing conditions, storage, food preparation, and cooking methods [65]. For example, in one study of the salicylate content of foods, an unpeeled pink lady apple had a salicylate content of 9.0 mg/kg, while a peeled pink lady apple had a content of 2.9 mg/kg [65]. This indicates that a significant portion of the salicylate content is located in the skin, an important observation for clinical practice. Other factors are likely to alter the food chemical content, such as length of time left in storage and cooking times.

Similar to ‘The Elimination Diet’ other diets low in food chemicals have been proposed and used worldwide although they are poorly studied. For example, ‘The Feingold Diet’ originally designed in the 1970’s to reduce attention deficit hyperactivity disorder (ADHD) in children eliminated artificial sweeteners, colours and preservatives from the diet but one re-challenge study using food colouring was unable to show an effect [66]. The ‘Few Foods diet’ excludes all foods except five or six (e.g. lamb, potato, rice, one of the brassicas, pear and tap water) but was shown to be ineffective at reducing refractory atopic dermatitis in children in one study [67]. Clearly more studies are needed to evaluate the use of such diets, both on their efficacy at reducing symptoms (gastrointestinal and extra-intestinal) as well as their feasibility and ability to maintain nutritional adequacy during implementation. Unfortunately the lack of studies assessing different diets low in food chemical content have likely been impeded by challenges in designing adequately controlled placebo-controlled studies, difficulty in obtaining funding to undertake expensive placebo-controlled feeding studies, and challenges in ethical issues related to risks of re-challenge studies in patients who could have potential fatal reactions such as those in steroid dependent asthma. With a surge in research and community interest in the role of diet for treatment of disease, funding may become easier to access and promote further dietary research within this area. 

#### 2.4.1. Food Additives and Chemical Induced Response

Prevalence of self-reported symptoms to food additives has been reported to be 0.01–0.23% in adults [68], and challenge studies have found 2–7% prevalence in atopic children [69]. The limited evidence comes mostly from case studies or small poorly controlled challenge studies, conducted in patients with asthma or chronic idiopathic urticaria/angioedema [70]. Food additives and chemicals are thought to contribute to both GI symptoms similar to IBS, as well as extra-intestinal symptoms including urticaria, headache, eczema, rhinitis, nasal congestion, or post nasal drip [71]. No controlled dietary trials have been published using the low chemical diet in its entirety in patients with GI disorders and limited data exists for other symptom responses. Small studies have investigated the effect of singular food chemicals. One small study of patients with a dual diagnosis of fibromyalgia and IBS, showed that a 4-week dietary exclusion of glutamate reduced >30% of symptoms in 84% of the patients, and symptoms were evoked by 3-day re-challenge [72]. The prevalence of GI symptoms following consumption of higher doses of salicylates from aspirin and other non-steroidal anti-inflammatory drugs has been suggested to be between 10-20% in those with asthma, and 0.6-2.5% in the general population [73]. However, it is unknown how this translates to the relatively lower doses found through dietary sources alone. It is also unknown if hypersensitivity to one food chemical predisposes an individual to be sensitive to other food chemical types.

#### 2.4.2. Proposed Mechanisms of Food Additives and Chemicals

Due to the lack of controlled studies, mechanisms of action of food additives in symptom provocation are poorly understood. It is likely that studies of each individual food additive will be required to elucidate their specific pathophysiology, few studies have been conducted to date with some suggestions of potential mechanisms of individual food additives. Inconsistent reports of adverse effects exist for tartrazine (food dye) [74], sulphites [75], benzoic acid [76] and monosodium glutamate [77] limiting reproducible evidence for mechanistic understanding. For most of these additives, several mechanisms have been proposed including IgE-mediated reactions and mitochondrial enzyme deficiencies. Specific mechanisms have been proposed to cause asthmatic reactions to sulphites with mode of exposure likely to be an important factor with inhalation of sulfur dioxide and the warm acidic environment of the mouth possibly triggering respiratory symptoms. Additionally, it has been proposed that the parasympathetic system may be involved whereby inadequate sulphite oxidase results in accumulation of sulphite, causing cholinergic mediated bronchoconstriction [78]. More recently, evidence from rat model studies has shown two commonly used emulsifiers, carboxymethylcellulose and polysorbate-80, can induce low grade inflammation and obesity/metabolic syndrome in wild-type hosts and may impair the epithelial barrier [79,80]. Similarly, nanoparticles such as titanium dioxide, a substance added to foods as a whitening agent, has been shown to alter nutrient absorption and disrupt the epithelial barrier impairing gut homeostasis in pre-clinical models at relevant exposure doses [45,81,82]. It has been proposed that food chemicals may induce a non-specific antigen-induced pseudo-allergic hypersensitivity [64,83]. Clearly more data is required to elucidate specific mechanisms of action of sensitivities to specific food additives.

#### 2.4.3. Diagnosis and Management of Food Additive and Chemical Sensitivity

There are no diagnostic tests available to assess food additive or chemical sensitivity. Despite the lack of efficacy data, the low chemical diet is used in clinical practice [71]. Like other exclusion diets, the low chemical diet is used as an initial short-term (2–6 week) restriction followed by strategic re-challenge to assess tolerance to each food chemical. Each food chemical is typically re-challenged in gradually increasing doses over a 3-day period [73]. Some chemicals such as dietary salicylates require challenging over longer periods (i.e., up to 10 days), due to the potential for a gradual build-up over several days that may be required to elicit a symptom response. Due to the number of food chemicals, the re-challenge process can be lengthy and result in extended use of the low chemical diet [71]. Concerns have been raised regarding the restrictive nature of the diet and potential negative consequences for nutritional adequacy, especially when used in pediatric patients [84], and as such it is key that a patient is supervised by a dietitian during the initial restriction as well as the re-challenge phases.

## 3. Genetic

### 3.1. Sucrose and Starch (Sucrase-Isomaltase Deficiency)

During normal carbohydrate digestion, sucrase cleaves the 1-4 linked glucose oligomers of sucrose and maltose, while isomaltase cleaves branched (1-6 linked) α-limit dextrins. Following this enzymatic digestion, the monosaccharides are absorbed across the epithelial barrier [85]. When there is an absence or reduction in sucrase and isomaltase, dietary carbohydrates such as sucrose and starches may result in symptom induction due to the inability or reduced ability for absorption. 

#### 3.1.1. Sucrase-Isomaltase Deficiency Response

In sucrase-isomaltase deficiency, sucrose and starches present in the lumen act as FODMAPs resulting in symptoms of diarrhea, bloating and abdominal pain, with symptom severity dependent on residual sucrase and isomaltase activity [85].

#### 3.1.2. Proposed Mechanisms of Sucrase-Isomaltase Deficiency

Sucrase-isomaltase deficiency may occur either due to genetic variants, or as a secondary or acquired event. There have been more than 25 genetic variants identified in the human sucrase gene. Variants can occur on the sucrase or isomaltase subunits, and as such varied degrees of symptom severity occur [85]. Secondary sucrase-isomaltase deficiency is usually transient and can be reversed with treatment of the primary condition. For example, studies in pigs have shown that villous atrophy such as that seen in coeliac disease, may result in sucrase-isomaltase deficiency. It has been estimated that 2–9% of those of North American or European decent may be affected by genetic variants, but the prevalence of secondary deficiency is not known [85]. A Swedish study of 250 patients found those with genetic sucrase-isomaltase variants were predisposed to IBS [86]. 

Regardless of the cause of sucrase-isomaltase deficiency, the absence or deficiency in enzyme activity results in undigested sugars in the lumen which then act as FODMAPs. The presence of undigested sugars in the lumen has osmotic effects resulting in hyperosmolar diarrhea, and subsequent fermentation in the colon results in gas production [85]. The distention leading from the increased water delivery and gas production in the lumen results in symptoms genesis. 

#### 3.1.3. Diagnosis and Management of Sucrase-Isomaltase Deficiency

The gold standard for diagnosis is duodenal or jejunal biopsies, although studies to date have largely been conducted in children [85]. Inaccuracies in diagnosis are likely due to the complexities in sample collection and storage that are required for analysis of sucrase, lactase, isomaltase and maltase activity [87]. Due to the invasive nature of obtaining biopsies, alternative methods have been suggested include sucrose breath testing, genetic sequencing which should be considered in combination with the clinical picture, or use of dietary restriction followed by oral enzyme replacement trials [85,87]. 

Dietary treatment may include restriction of sugars and starch, followed by gradual re-introduction to determine tolerance, although compliance rates are poor and efficacy of dietary modification have not been well studied [87]. An alternative or adjunct therapy would be the use of enzyme replacement therapy with sacrosidase, which has shown good effect in small studies [87,88]. 

## 4. Discussion

### 4.1. Recommendations for Future Research

Double-blind placebo-controlled with cross-over trials are the current gold standard for confirming the dietary factor(s) involved in symptom generation and are therefore required to confirm any food intolerance discussed in the current review. Greater methodological difficulties exist in the design and implementation of clinical dietary trials compared to pharmacological trials. For example, lack of isolation of food components e.g. co-existence of gluten and fructan within the same foods, difficulty in design of placebo diets, and challenges in blinding of participants. Hence, the same criteria for trial methodology used in pharmacological studies may not be appropriate [89]. In order to ensure well designed dietary trials can be conducted, adequate food compositional data is required. Food compositional data is currently available for a range of foods containing gluten, wheat and FODMAPs. However, further food compositional data is required for other potential problematic food components such as histamine and food chemicals. 

Once food compositional data is available and clear efficacy has been established, further research should investigate the following key factors. Firstly, assess the role of mode of education on symptomatic response i.e., does the efficacy vary depending on how the information is delivered, either via physician, dietitian, nurse, or online and app-based mediums. Secondly, what is the level of dietary restriction required to achieve symptom resolution. For example, a strict gluten-free diet is required in coeliac disease to ensure mucosal healing, but it is unknown if this same level of restriction be necessary for those with NCG/WS to reduce symptoms. Likewise, feeding studies assessing efficacy of the low FODMAP diet reduced intake to 3 g/day [4], compared to dietary education trials achieving 10–12 g/day with similar levels of efficacy achieved [16,17]. Finally, assessment of the long-term outcomes and safety implications are required. As many efficacy trials will end after an initial restriction period, little is known about the long-term dietary requirements, effect on nutritional profiles and quality of life, as well as effect on microbiota and metabolomic profiles. 

The ability to predict response to dietary therapies would significantly enhance treatment strategies which has been reviewed previously [90]. Prediction of response would allow clinicians to tailor which therapy is used, and avoid potential negative implications of dietary restrictions such as reduced nutritional adequacy and alterations to the microbiome. Unfortunately non-invasive techniques such as hydrogen breath testing for carbohydrate intolerances lack reproducibility and are not recommended [91]. Hence, recent studies have investigated alternative strategies to predict response such as faecal profiling of microbiota [92,93] and volatile organic compounds [94] or urinary metabolomics [27]. However, despite increased research interest, ability to predict response is still in its infancy and is far from implementation into practice. 

The ability to identify ways to predict response to dietary therapies may also lead to improved mechanistic understanding of functional GI conditions and allow for creation of ways to improve tolerance to foods. For example, certain microbiota or metabolomics profiles may indicate that supplementation with a certain pre- pro- or syn-biotic is required, shifting the profile to one in which symptom response to a certain food component is mitigated. 

### 4.2. Implications and Recommendations for Clinical Practice

Due to the lack of tests available to identify intolerance to specific food components, a trial-and-error approach is used in clinical practice. This approach utilizes strategic short-term removal of food components followed by re-introduction to assess symptom response. Selection of which food components to trial removing may be based on the clinical picture including symptomatic profile, habitual diet, patients suspicions of food intolerances, and where applicable, genetic variations. 

Appropriate implementation and education about avoidance of food triggers is required across any food intolerance (and allergy). Patients attempting to modify their diet benefit from guidance by a dietitian, likely due to improved understanding and compliance, resulting in greater symptom response [30,32,95]. The role of the dietitian is important not only during the initial stages of establishing the dietary modification whereby the dietitian can modify the level of restriction required based on the clinical picture; but is also integral in the implementation and interpretation of re-challenges to assess tolerance to specific food triggers [31]. Appropriate education also ensures patients do not over-restrict their diet unnecessarily or undertake ‘diet-stacking’ in which they accumulate multiple dietary restrictions resulting in worsened food variety and nutritional adequacy. 

In some cases, combination therapies may allow for more personalized and targeted therapies. For example, in a re-analysis of patients within a trial assessing the low FODMAP diet, patients who were carriers of the sucrase-isomaltase hypomorphic variants were less likely to respond to a 4-week low FODMAP diet trial [96]. In these patients, sucrose and starch restriction alone or in combination with a low FODMAP diet may have been more efficacious. Likewise, implementation of simultaneous probiotic supplementation may mitigate any potential negative consequences of the low FODMAP diet on the microbiota [16]. Additionally, strategies to improve tolerance to specific food components may reduce the level of dietary restriction necessary such as the use of α-galactosidase in patients who do not tolerate galacto-oligosaccharides [97]. 

## 5. Conclusions

It is clear that while there are a large number of suspected food components inducing symptoms, there is a notable shortage of well-designed studies making understanding of the reaction, diagnosis and management difficult. Future studies should use carefully planned double-blind studies with defined inclusion criteria to reduce the knowledge gaps and elucidate clear mechanisms of action of food intolerances. 

## Figures and Tables

**Table 1:** Foods containing histamine [56].

**Meat**	Sausages of any kind, salami, air-dried and corked meat, ham, etc.
**Fish**	Dried or preserved fish, such as herring, tuna, mackerel, sardines and anchovies, seafood, fish sauces.
**Cheese**	All types of hard, soft and processed cheese
**Vegetables**	Eggplant, avocado, sauerkraut, spinach, tomatoes incl. tomato juice/ketchup
**Drinks and liquids**	Vinegar or alcohol of all kinds, mainly red wine, beers, champagne, whisky and cognac; alcohol in general reduces degradation of histamine and increases the permeability of the intestine and can therefore worsen the symptoms of histamine intolerance in general

**Table 2:** Diagnosis of histamine intolerance.

**Diagnostic criteria [58]**	
The diagnosis of histamine intolerance is made by a combination of the following criteria: presenting ≥2 typical symptoms of histamine intolerance (see below) improvement through histamine-free diet improvement through antihistaminergic medication.	
**Symptom types [57,58]**	
Skin	Itching, sudden reddening of the skin (flush symptoms) on the face and/or body, very rarely hives, angioedema (different to urticaria) and other exanthemas
Digestion	Nausea, vomiting, diarrhea, abdominal pain
Circulation	Tachycardia, drop in blood pressure, dizziness
Respiratory	Chronic nasal flow, sneezing attacks
Neurological	Headaches, migraines
Gynecological	Menstrual cramps

**Table 3:** Examples of foods containing natural and added food chemicals thought to induce gastrointestinal and extra-intestinal symptoms in gastrointestinal conditions.

	FOOD CHEMICAL	FOOD SOURCES [64,65]
**Natural food chemicals**	Amines	Cheese, chocolate, banana, ham, fish
	Glutamate	Tomato
	Salicylates	Apples, tomatoes
**Added food chemicals**	Antioxidants	Oils, margarine
	Benzoates	Soft drinks, cordials
	Colors	Confectionary, jelly
	Monosodium glutamate (MSG)	Chinese take-away, packaged foods
	Nitrates	Deli meats
	Propionates	Breads
	Sorbic acid	Processed cheese slices
	Sulfites	Soft drink, cordials, dried fruit
